# Evaluating a partnership model of hospice enabled dementia care: A three-phased monitoring, focus group and interview study

**DOI:** 10.1177/02692163221116763

**Published:** 2022-09-05

**Authors:** Dorry McLaughlin, Felicity Hasson, Joanne Reid, Kevin Brazil, Lesley Rutherford, Carol Stone, Jenny T van der Steen, Joanne Ballentine

**Affiliations:** 1 School of Nursing and Midwifery & Centre for Evidence and Social Innovation, Queen’s University, Belfast, Northern Ireland; 2 Institute of Nursing and Health Research, Ulster University, Shore Road, Newtownabbey, Northern Ireland; 3 Belfast Health and Social Care Trust, Marie Curie Hospice, Belfast, Northern Ireland; 4 Department of Public Health and Primary Care, Leiden University Medical Center, Leiden, The Netherlands; 5 Department of Primary and Community Care, Radboud University Medical Center, Nijmegen, The Netherlands; 6 Northern Ireland Hospice, Belfast, Northern Ireland

**Keywords:** Hospice, dementia, caregivers, interview, focus groups

## Abstract

**Background:**

People with dementia and their caregivers often lack equitable access to hospice care which is a concern internationally. Domains of best practice in palliative care for this population exist and hospices are urged to become dementia friendly.

**Aim:**

This study aimed to evaluate the model of ‘Hospice Enabled Dementia Partnership’ mapped to international domains of best practice.

**Design:**

Three-phased monitoring, group interview and individual interview study using a formative evaluation framework.

**Setting**/**Participants:**

The partnership model was a collaboration between a large specialist palliative care hospice, a dementia charity and a Health Care Trust in the United Kingdom. Service documents were subjected to documentary review of monitoring activity and key indicators of service success. Group interviews and individual interviews took place with family carers (*n* = 12), health care professionals involved in delivering the service (*n* = 32) and senior professionals (*n* = 5) responsible for service commissioning in palliative or dementia care.

**Results:**

One hundred people with dementia were referred to the service between May 2016 and December 2017. Thirty-eight of the 42 people who died, achieved their preferred place of care and died at home. Four themes were derived from the data ‘Impact of Dementia’, ‘Value of the Service’, ‘Information and Learning Needs’ and ‘Working in Partnership’.

**Conclusions:**

Positive outcomes resulted from this best practice model; achievement of preferred place of care and death at home, dual benefits of therapies for patients and families and partnership in cross working and learning between services. Replication of this model should be considered internationally.

## Key statements

**What is already known about the topic?**

- Concerns have been highlighted across the globe around unmet needs at the end of life of people with dementia and their caregivers
- Although positive outcomes from access to hospice care for people with dementia and their caregivers are evidenced there is also evidence that access to hospice care is suboptimal.
- International (EAPC) domains of best practice in palliative care of people with dementia exist to guide services in caring for this population and their caregivers.

**What this article adds?**

- This study presents a model of palliative care for people with dementia and their caregivers, mapped to internationally agreed-upon domains of optimal palliative care for this population. This provides outcomes such as achievement of preferred place of care and death at home and dual benefits of therapies for patients and family carers.
- The study provides insights into the delivery of a dementia friendly partnership across hospice and community settings, which reflects the WHO ethos of age-friendly initiatives. A reciprocal partnership relationship in cross working and learning between services who lack a history of working together has been generated.
- This is an evidence-based example and framework for use internationally by other services wishing to implement such dementia-friendly initiatives. This would require consideration of dementia awareness and dementia palliative care training for staff, creation of new working service partnerships and possible adaptations to the environment such as building design, signage, lighting and colour contrast.

**Implications for practice, theory or policy?**

- The study also promotes the concept of a public health model for palliative and dementia care
- Given these experiences and given the international relevance and incidence of dementia globally, this partnership model of palliative care for people with dementia should be replicated

## Introduction

Dementia is recognised as a major health issue. Globally 55 million people have this condition, which is projected to increase to 139 million by 2050.^1^ Dementia is an incurable condition progressively limiting the lives of those diagnosed.^2,3^ The concept of dementia- friendly has originated to promote age-friendly communities,^4^ with various initiatives^5^ such as dementia awareness training, and consideration of the building design and architecture-.^6,7^ This refers to the physical and social environment promoting inclusion, acceptance and access for people living with dementia and family caregivers.^7–10^

Internationally palliative care is recognised as relevant to people with dementia.^11^ Concerns exist around unmet end-of-life care needs for people with dementia and family caregivers^12–14^ and healthcare needs to be tailored to their needs. Many dementia- friendly initiatives have focussed on acute care, yet the condition is incurable, and end- of- life care is sub-optimal.^15,16^

Across the world, a number of different models of hospice and specialist palliative care exist.^17^ These can include hospice and specialist palliative care teams providing care to in-patients and outpatients in hospice or hospital, and to those in day care, community, and nursing home settings.^17^ Internationally hospice care is infrequently accessed by people with dementia and family caregivers,^12,18,19^ but studies from America show an increased number of this population are being referred to hospice care.^20–23^ Hospice care can create desirable outcomes such as achievement of preferred place of care and death and caregivers reporting positive experiences of care.^24–26^ This is important given that family caregivers often provide most of the care to their loved one but may feel unequipped.^27–30^

Models of specialised palliative care input for people with advanced dementia, in the Netherlands, included a palliative care unit delivering specialised care to those with advanced dementia in a nursing home^31^ Studies in hospices in New Zealand, America and United Kingdom have developed end-of-life care for people with dementia using educational approaches for staff^32–34^ whilst one initiated a hospice and dementia charity partnership.^34^

The concept of Hospice Enabled Dementia Care has been promoted in the United Kingdom^19^ and also at a wider European level.^11,19^ Central to this concept are building new skills, establishing new service partnerships and creativity in the provision of care to meet the needs of people with dementia.^19^ Hospice Enabled Dementia Care^19^ promotes and resonates with the European Association for Palliative Care international domains of evidenced best practice in dementia care.^11^ Eleven care domains were recommended which services should address. Previous studies have developed care relevant to a small number of these domains such as staff education.^32–34^

Against this backdrop, of unmet needs of people with dementia at end of life, a Hospice Enabled Dementia Partnership, specifically mapped to the eleven international domains of best practice^11^ has been implemented in the United Kingdom. This partnership exists between a large specialist palliative care hospice, a leading United Kingdom dementia charity (providing advice, support and resources to people with dementia and their carers) and community services in palliative and dementia care within a Health Care Trust. This project, as an intervention, is described using the TIDieR Checklist^35^ recognised as applicable across all evaluative study designs (Supplemental Appendix 1). The Hospice Enabled Dementia Partnership offered: Holistic assessment and care of the person with dementia and family carers, and assessment and management of symptoms, behaviours and the environment. This was provided through partnership working between palliative care and mental health/ dementia services in a Dementia- Friendly Hospice or in the person’s home. Staff had developed enhanced knowledge and skills in dementia palliative care.^35^

### Aim

This study aimed to evaluate the model of Hospice Enabled Dementia Partnership, mapped to international domains of best practice, between a large specialist palliative care hospice, a United Kingdom dementia charity and a Health Care Trust.

## Methods

### Design

**Figure 1.**
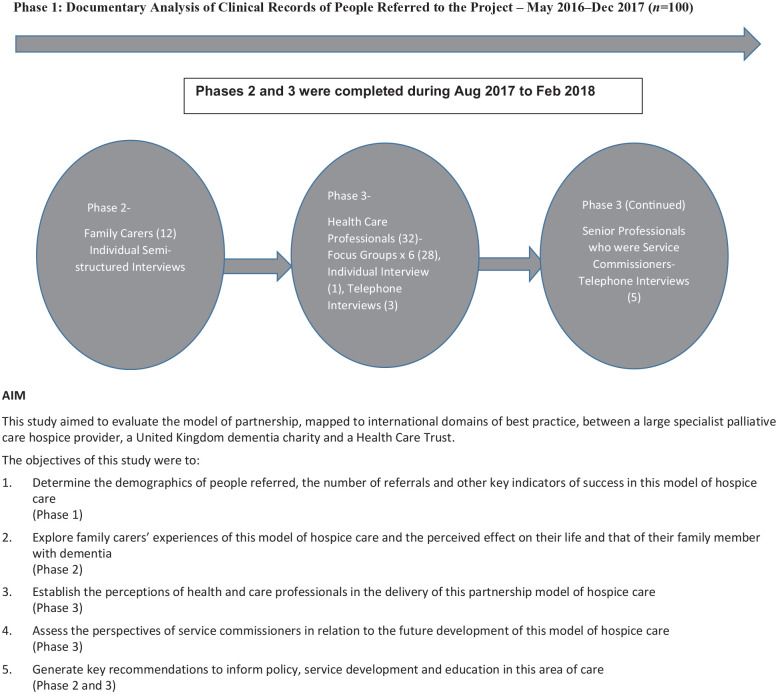
Three phases of the study.

This study used a three-phase, formative evaluation framework to identify influences and study the complexity of the implementation^36^ of a novel Hospice Enabled Dementia Partnership model of care. The three phases are shown in Figure 1. Phase 1 involved documentary analysis of clinical records of people with dementia referred to the project. Semi-structured individual, face-to-face interviews took place in Phase 2 with family carers of those referred. In Phase 3 health care professionals involved with the project took part in focus group interviews and some requested an individual or telephone interview. Phase 3 also involved telephone interviews with senior professionals responsible for service commissioning in palliative or dementia care. A qualitative approach was included in this study to obtain rich data, through focus groups and interviews, around participants’ experiences and perceptions of the Hospice Enabled Dementia Partnership. Consolidating criteria for reporting qualitative research (COREQ) guidelines^37^ were used.

### Population

The population under study were patients and their family carers referred to the Hospice Enabled Dementia Partnership, health care professionals who were involved in the project and senior professionals regionally responsible for service commissioning in palliative or dementia care.

#### Criteria

Inclusion criteria for individual, face-to-face interviews with family carers were that they:

- Were 18 years old or over
- Had been or were caring for a family member with dementia referred to the Hospice Enabled Dementia Partnership project
- They were excluded if they had experienced a recent bereavement within the previous 3 months^38,39^

Inclusion Criteria for focus groups with health care professionals were that they:

- Had cared for people with dementia within the Hospice Enabled Dementia Partnership Project

Inclusion criteria for individual telephone interviews with service commissioners were that they:

- Were senior professionals responsible for service commissioning in palliative care or dementia care

All participants provided written informed consent.

### Setting

This study took place within a partnership of a large specialist palliative care hospice, a Dementia Charity and a Health Care Trust. Interviews with family carers took place either in the Dementia Day Hospice or their own home. A private room at the hospice was used for focus groups and interviews with health care professionals. Telephone interviews with service commissioners took place in a private room at the University.

### Recruitment and data collection

Purposive sampling was used with all participants who met the inclusion criteria.

Phase 1. The project clinical lead of the Hospice Enabled Dementia Partnership provided anonymised data from clinical records of each person with dementia referred to the project during May 2016–December 2017. This determined nature of referrals, age range, length of time in care, place of care and death. Patient outcomes collected included; recorded advance care planning discussions, expressed advance care planning preferences, achievement of preferred place of care and death, number of best interest decisions,^40^ number of unscheduled hospital admissions and record of anticipatory prescribing for out of hours care.

For Phases 2 and 3, a letter about the study was forwarded to potential participants by the project clinical lead. Those interested contacted the Principal Investigator of the study directly and were forwarded an information and recruitment pack. All were eligible and interviews took place at an arranged place, date and time following receipt of a signed consent form. Interviews and focus group guides, containing broad questions and prompts, were informed by the international domains of best practice for palliative care of people with dementia^11^ (Supplemental Appendix 2). They lasted for 40–80 min and all participants were provided with information on support and counselling services.

Semi-structured, face-to-face interviews took place in Phase 2 with 12 family carers of those referred. Nine were interviewed in a private room within the Dementia Day Hospice and three at home. At the time of data collection none of the patients referred to the project had capacity to consent nor to be interviewed. In Phase 3, health care professionals involved with the Project took part in six focus group interviews (*n* = 28), at their request three participants, unable to attend the focus group, took part in individual telephone interviews and one other in a face-to-face interview.

Phase 3 also involved five individual telephone interviews with senior professionals regionally responsible for service commissioning in palliative or dementia care. Twelve were invited to take part in a telephone interview, but only five responded. Data Collection for Phases 2–3 took place between August 2017 and February 2018.

### Data analysis

Quantitative data (Phase 1) were analysed using descriptive statistics. Qualitative data (Phases 2 and 3) were subjected to thematic analysis by DMcL, FH, JR and LR, using a recognised six-step framework (familiarisation, coding, generating themes, reviewing themes, defining and naming themes, writing up).^41^ Transcripts were independently analysed by three other team members (KB, CS and JS) and themes agreed on discussion.

### Ethical considerations

Ethical approval was obtained from NHS Research Ethics Committee Reference Number: 17/SS/0024 in April 2017.

## Results

### Phase 1: Analysis of clinical records of people referred to the project

One hundred people with dementia were referred to the project from May 2016 to December 2017. All but seven (7%), referred for caregiver support or respite care, were referred for either end-of-life care (*n* = 41, 41%) or holistic symptom assessment and management (*n* = 52, 52%) (Table 1). Patient characteristics are shown in Table 2. Of 82 people (82%) with a single dementia type specified, 65 (65%) had Alzheimer’s disease (Table 2). One quarter of people referred (*n* = 25, 25%) did not have any recorded co-morbidity.

**Table 1.** Nature of referrals (*n* = 100).

Main reason for referral	Number
Care in last days of life	*n* = 41 (41%)
Holistic assessment	*n* = 24 (24%)
Pain and symptom assessment and management	*n* = 28 (28%)
Carer support	*n* = 6 (6%)
Respite care	*n* = 1 (1%)
Total	*n* = 100 (100%)

**Table 2.** Characteristics of patients (*n* = 100).

Demographics	
Gender	
Women	*n* = 55 (55%)
Men	*n* = 45 (45%)
Ethnic origin	All White
Age Mean (SD) [Range], years	82 (9) [49–100]
Dementia Diagnosis	**Number and %**
Alzheimer’s disease	*n* = 24 (24%)
Probable Alzheimer’s disease	*n* = 41 (41%)
Vascular dementia	*n* = 11 (11%)
Mixed aetiology dementia	*n* = 8 (8%)
Lewy body dementia	*n* = 4 (4%)
Parkinson’s dementia	*n* = 1 (1%)
Frontotemporal dementia	*n* = 1 (1%)
Unspecified dementia	*n* = 10 (10%)
Co-morbidity	**Number and %**
Cardiac disease	*n* = 19 (19%)
Cancer	*n* = 15 (15%)
Glaucoma	*n* = 1 (1%)
Parkinson’s disease	*n* = 4 (4%)
Chronic kidney disease	*n* = 2 (2%)
Depression	*n* = 2 (2%)
Osteoarthritis	*n* = 9 (9%)
Osteoporosis	*n* = 2 (2%)
Chronic obstructive pulmonary disease	*n* = 3 (3%)
Type 2 Diabetes mellitus	*n* = 4 (4%)
Cerebral vascular accident	*n* = 3 (3%)
Diverticular disease	*n* = 2 (2%)
Peptic ulcer	*n* = 1 (1%)
Pulmonary fibrosis	*n* = 2 (2%)
Fibromyalgia	*n* = 2 (2%)
Asthma	*n* = 3 (3%)
Pernicious anaemia	*n* = 1 (1%)
No recorded co-morbidities	*n* = 25 (25%)

Forty-two people with dementia (42%) died during this timeframe, 38 (38%) at home and four (4%) in hospital admitted there from their own home. The median length of time these 42 people were cared for was 22 days. Family caregivers reported preferred place of care and death as achieved for all 38 (38%) people who died at home. Advance care planning took place with 22 people with dementia (22%) and preference outcomes recorded. Anticipatory prescribing for out of hours care took place with the majority of people referred (*n* = 96, 96%).

### Phases 2 and 3: Interviews and focus groups on experiences of the project

Table 3 shows the demographics of interviewees. They were predominantly women and all white ethnicity. Professionals were representative of the inter-disciplinary team, in both palliative care (*n* = 27) and mental health (*n* = 5). Four core combined themes were derived from the data: ‘Impact of Dementia’, ‘Value of the Service’, ‘Information and Learning Needs’ and ‘Working in Partnership’.

**Table 3.** Demographics of family carers, health care professionals and service commissioners.

Family carers (*n* = 12)		
Gender	Women	*n* = 11 (92%)
	Men	*n* = 1 (8%)
Age range in years	41–50	*n* = 4 (33%)
	51–60	*n* = 2 (17%)
	61–70	*n* = 3 (25%)
	Over 70	*n* = 3 (25%)
Ethnic origin	White	*n* = 12 (100%)
Caring role	Full-Time	Ranged from 1 to 15 years
Health care professionals (*n* = 32)		
Gender	Women	*n* = 29 (91%)
	Men	*n* = 3 (9%)
Age range in years	20–29	*n* = 2 (6%)
	30–39	*n* = 6 (19%)
	40–49	*n* = 11 (34%)
	50–59	*n* = 7 (22%)
	60–69	*n* = 6 (19%)
Ethnic origin	White	*n* = 32 (100%)
Professional role	Nursing	*n* = 18 (56%)
	Medicine	*n* = 3 (9%)
	Social Worker	*n* = 2 (6%)
	Physiotherapist	*n* = 2 (6%)
	Occupational Therapy	*n* = 3 (9%)
	Chaplaincy	*n* = 1 (3%)
	Other	*n* = 3 (9%) (Complementary Therapy & Dementia Support Workers)
Time in current professional role		Ranged from 1 to 15 years
Service commissioners (*n* = 5)		
Gender	Women	*n* = 3 (60%)
	Men	*n* = 2 (40%)
Age range in years	41–5051–6061–70	*n* = 1 (20%)*n* = 3 (60%)*n* = 1 (20%)
Ethnic origin	White	*n* = 5 (100%)
Time in current professional role		Ranged from 1 to 10 years

### Impact of dementia

Across the data, the reported impact of dementia on the person, family and daily living was apparent and relevant to the support and help which the Hospice Enabled Dementia Partnership offered. (Supplemental Appendix 1)

‘*He can be very aggressive. He did attack me one night. I had to ring my family at 3.30 in the morning screaming and crying ‘Come and help me’ (Family Carer, 8).*

There was a range of symptoms which health professionals had observed and which required assessment and management:

‘*The big ones that we would see would be restlessness, agitation and depression is a big co-morbid problem with dementia. . .other ones that we get referred to us- aggression*, physical and verbal aggression’ (Health care professional 31).

Pain as a symptom could be underdiagnosed:

‘*Most of the patients referred, I would say about 85%, have had some aspect of pain even if that hasn’t been on the referral sheet, because pain would be one of the most underdiagnosed symptoms that somebody with dementia has. . .not understanding that what they feel is pain. . .not being able to express it’ (Health care professional 29)*

In addition to carer burden support for family carers was required in coping with the loss of the person they had known:

*‘As the illness progresses you start to lose sight of the person that they once were. I would sometimes say. . .it robs the sufferer of their memories; it also robs the carers, because I try to get old photographs out, but I can only remember X as X now. I find it very difficult to remember the old X’ (Family Carer 6).*

### Value of the service

The Dementia Friendly Day Hospice, compared to other day care, was perceived unique in offering dual holistic support and therapies to both the person and family caregivers.

*‘We have an aromatherapist here-the carers get aromatherapy as well which is lovely. . . and each week one of the staff nurses will come along and invite me in to have a little chat and see if there is any deterioration in X’s condition or if there is anything that they could do to help. . .then they would inform the appropriate service. . .honestly, they’re brilliant’ (Family Carer 1)*

Key outcomes for family caregivers were peer support, being with others and socialisation. This peer support network helped in sharing concerns, problems and fears and provided insights into the care journey. It was a safe place and they appreciated emotional support from staff:

*‘It’s a wonderful service (Day Hospice) you don’t think the staff are watching, but they are and they can see when someone is upset, the staff they’ll put their arm around you so they’re making you feel like a person (Family Carer, 6).*

Positive feedback was also echoed by professionals who referred people to the Hospice Enabled Dementia Partnership:

*‘I think it’s been a very positive project. . . the feedback I get from patients and their relatives. The Day Hospice. . .the patients and carers that I know who go to that . . .they absolutely love it, not only from the patient’s point of view, but the carers get so much out of it as well’ (Health care professional 31)*

Some family caregivers found it difficult seeing other people with dementia, but valued observing their family members engaging with therapy and receiving stimulation:

*‘Quite a lot of it is music and music is good for the brain and I can see X coming out of himself and taking part and doing physical movements in time with the music and really taking an interest in what’s happening’ (Family Caregiver 1)*

Similarly, professionals observed the benefits of therapies in linking with the person with dementia thus seeing the person behind the illness.

*‘Creative therapy . . .it’s mainly to stimulate them and see where they feel comfortable . . .bring them together and do a one-to-one session as well because Creative Therapy can bring them out of the dementia and see the person for who they are’ (Health care professional 2.*

It was also apparent that the service placed emphasis on providing person centred care:

*‘Even though someone’s got dementia, it’s like a label, but they’re still a human being. . .Your still the person. . .I don’t think us as a team, we don’t look at any of our patients as. . .the condition. . .it’s the person first’ (Health care professional 1).*

Family caregivers valued the person with dementia being accepted and felt this addressed isolation:

*‘Now x comes from a big extended family and they sort of avoid him. It’s just been so isolating. . . every so often he’ll sing a couple of words and then he’ll look around him. Everybody (Day Hospice) just accepts them because everybody is in the same position’ (Family Caregiver 9)*

Family caregivers reported positive experiences of home visits and telephone contact in providing emotional support and advice, thus reducing isolation and in use of therapies for symptom and behavioural management and comfort:

*‘X comes out and she does Mummy, her feet and all the oils to help her breathe and it’s fantastic. Mummy loves it and she plays her music and Mummy just lights up when she comes. The oils they’re lovely and they soothe her because she gets lovely wee sleeps after it’ (Family Carer 11).*

Professionals and Service Commissioners highlighted the need for the project to be available to people with dementia over a wider geographical region to ensure equity of service access.

### Information and learning needs

All participants understood palliative care and the project highlighted the applicability of palliative care to non-malignant disease. Most family caregivers had self-sought information from the internet. Several had completed a Dementia Charity’s Informal Carer Course and read their leaflets and some identified their own learning needs around the dementia disease and advance care planning.

Professionals perceived the practical management of people with dementia to be a learning need for family caregivers. All family caregivers perceived staff to be competent and skilled. Staff attended a Dementia Awareness Day or completed a Certificate Course in Holistic Dementia Care. They identified a number of other specific training areas: education of primary care teams for early identification of people with dementia; recognition of dementia as a palliative care condition; timely advance care planning and behavioural assessment and management. A key area of learning was identified:

‘*One of the successes. . .the legacy that’s left behind is that there’s an understanding about palliative care within the mental health teams and there’s an understanding about mental health dementia care within the palliative care teams and to me that’s been very good’ (Health care professional 29)*

Collaborative working generating shared learning and expertise between services was valued:

*‘I know some of the psychiatric nurses would have said . . .they would be familiar with recognising somebody’s behaviour and when it’s deteriorating into moderate to severe dementia, but they wouldn’t always recognise palliative signs, whereas the palliative nurses would have said we can recognise the palliative signs, but we wouldn’t know the dementia signs’ (Service Commissioner 1)*

A public health approach about dementia and relevance of palliative care was suggested. Participants proposed education to disseminate knowledge and skills from the project. This included screening and identifying training needs,^42^ clinical placements across both services, recognised courses on palliative care and dementia and other modes of education.^43^

### Working in partnership

Family caregivers reported that staff included them in decision-making and care planning. They had observed partnership between staff where different uniforms and badges were worn. There was a professional consensus on the importance of partnership between mental health and palliative care services. Developing good relationships was pivotal to the success of the project:

*‘The first year of the project, building those relationships was probably one of the most important things. . .there was a genuine willingness for each world of mental health and palliative care, wanting to get this right for this patient population. I think if it wasn’t for the project’s creation, we wouldn’t have the partnerships now that we have and we didn’t have at the beginning of the project’ (Health care professional 16)*

A reciprocal partnership relationship in cross working between mental health and palliative care services was generated by the project:

‘*I think this project has been really invaluable in promoting the partnerships between ourselves and palliative care. People I have been worried about have been referred through and the project have been very, very quick to pick them up. Even just building personal relationships with people out of the hospice, it’s been very helpful and very positive. They know they can pick up the ‘phone and ring me if they want advice about something’ (Health care professional 31)*

Professionals recognised challenges and enablers to partnership working. Perceived challenges were time and role ambiguity whilst enablers were mutual respect for roles and ability to work as a team. Table 4 shows findings mapped to the International Domains of Best Practice for people with dementia The Key Findings of the study can be seen in Table 5.

**Table 4.** Evaluating a partnership model of hospice enabled dementia care: Findings mapped to European Association for Palliative Care International Domains of best practice for people with dementia (van der Steen et al, 2014).^11^.

Domain of Best Practice	Phase of the Study	Findings/ Quotations
D1: Application of Palliative Care	TIDieR (Supplemental Appendix 1) Description of the Hospice Enabled Dementia Partnership	**Evidence of palliative care/ specialist palliative care approach** ‘On the **1st assessment**, whether in the Dementia Friendly Day Hospice or at home, a detailed history was taken by the Project Lead, from both patient and carer to establish a specialist palliative care plan for intervention. These interventions could be for physical, social, emotional or spiritual symptom management and support’
D2: Person-Centred Care, Communication and Shared Decision Making D2 (Continued)	Phase 3- Focus Group with Health care professionals Theme: Value of the Service Phase 3- Focus Group with Health care professionals Theme: Value of the Service Phase 1- Anonymous Data from Clinical Records Phase 2- Interviews with Family carers Theme: Value of the Service	**The service placed emphasis on providing person centred care:** *‘Even though someone’s got dementia, it’s like a label, but they’re still a human being. . .You’ re still the person. . .I don’t think us as a team, we don’t look at any of our patients as. . .the condition. . .it’s the person first’ (Health care professional 1).* **Professionals observed the benefits of therapies in linking with the person with dementia thus seeing the person behind the illness.** *‘Creative therapy . . .it’s mainly to stimulate them and see where they feel comfortable . . .bring them together and do a one-to-one session as well because Creative Therapy can bring them out of the dementia and see the person for who they are’ (Health care professional 2).* **Communication and shared decision making** Twenty-two people with dementia (22%) admitted to the service took part in advance care planning, with the Project Clinical Lead of the Hospice Enabled Dementia Partnership and their preference outcomes were recorded. If the patient lacked cognitive capacity to take part in advance care planning, the best interest decision making process was discussed with the family carer. **Communication and shared decision making** *‘. . .each week one of the staff nurses will come along and invite me in to have a little chat and see if there is any deterioration in X’s condition or if there is anything that they could do to help. . .then they would inform the appropriate service. . .honestly, they’re brilliant’ (Family carer 1)*
D3: Setting care goals and advanced care planning	Phase 1- Anonymous Data from Clinical Records	**Advance care planning/ Setting care goals** Twenty-two people with dementia (22%) admitted to the service took part in advance care planning, with the Project Clinical Lead of the Hospice Enabled Dementia Partnership and their preference outcomes were recorded. If the patient lacked cognitive capacity to take part in advance care planning the best interest decision making process was discussed with the family carer Family carers reported preferred place of death as achieved for all thirty- eight people (38%) who died at home.
D4: Continuity of Care	Phase 2- Interviews with Family carers Theme: Value of the Service	**Continuity of care** *‘. . .each week one of the staff nurses will come along and invite me in to have a little chat and see if there is any deterioration in X’s condition or if there is anything that they could do to help. . .then they would inform the appropriate service. . .honestly, they’re brilliant’ (Family carer 1)*
D5: Prognostication and timely recognition of dying	TIDieR (Supplemental Appendix 1) Description of the Hospice Enabled Dementia Partnership	Prognostication tools were used such as: the Supportive and Palliative Care Indicator Tool SPICT-2-sided.pdf (oxfordshireccg.nhs.uk). For patients with dementia, referred to the Hospice Enabled Dementia Partnership, this tool helped to provide an indication of deteriorating health and clinical signs of advanced, progressive disease.
D6: Avoiding overly aggressive burdensome or futile treatment	TIDieR (Supplemental Appendix 1) Description of the Hospice Enabled Dementia Partnership	As reported in the TIDieR care and treatment, within the Hospice Enabled Dementia Partnership, was underpinned by a palliative care approach which focussed on comfort, quality of life, psychosocial and spiritual support rather than overly aggressive, burdensome or futile treatment.
D7: Optimal treatment of symptoms and providing comfort	Phase 2-Interview with Health care professional Theme: Impact of Dementia Phase 2- Interview with Family carer Theme: Value of the Service	**Undiagnosed pain was frequently present in those referred to the service:** ‘*Most of the patients referred, I would say about 85%, have had some aspect of pain even if that hasn’t been on the referral sheet, because pain would be one of the most underdiagnosed symptoms that somebody with dementia has. . .not understanding that what they feel is pain. . .not being able to express it’ (Health care professional 29)* **Use of complementary therapies for symptom management and comfort** *‘X comes out and she does Mummy, her feet and all the oils to help her breathe and it’s fantastic. Mummy loves it and she plays her music and Mummy just lights up when she comes. The oils they’re lovely and they soothe her because she gets lovely wee sleeps after it’ (Family carer 11).*
D8: Psychosocial and spiritual support	Phase 2-Interview with Family carer Theme: Value of the Service	**Psychosocial and spiritual support to carers** *‘It’s a wonderful service (Day Hospice) you don’t think the staff are watching, but they are and they can see when someone is upset, the staff they’ll put their arm around you so they’re making you feel like a person (Family carer, 6).* **Encouragement to family members re effect of therapies on their loved ones** ‘Quite a lot of it is music and music is good for the brain and I can see *X* coming out of himself and taking part and doing physical movements in time with the music and really taking an interest in what’s happening’ (Family carer 1)
D9: Family care and involvement	Phase 3- Interview with Health care professional Theme: Value of the Service	**Family care and involvement** *‘I think it’s been a very positive project. . . the feedback I get from patients and their relatives. The Day Hospice. . .the patients and carers that I know who go to that . . .they absolutely love it, not only from the patient’s point of view, but the carers get so much out of it as well’ (Health care professional 31)* *‘We have an aromatherapist here-the carers get aromatherapy as well which is lovely. . . and each week one of the staff nurses will come along and invite me in to have a little chat and see if there is any deterioration in X’s condition or if there is anything that they could do to help. . .then they would inform the appropriate service. . .honestly, they’re brilliant’ (Family carer 1)*
D10: Education of the Healthcare Team	Phase 3-Focus Group with Health care professionals Theme: Information and Learning Needs Phase 3-Telephone Interview with Service commissioner Theme: Information and Learning Needs	**A key area of learning from the project was identified:** ‘*One of the successes. . .the legacy that’s left behind is that there’s an understanding about palliative care within the Mental Health Teams and there’s an understanding about mental health dementia care within the palliative care teams and to me that’s been very good’ (Health care professional 29)* **The value of collaborative working generating shared learning and expertise between palliative care and mental health services was recognised:** *‘I know some of the psychiatric (mental health) nurses would have said . . .they would be familiar with recognising somebody’s behaviour and when it’s deteriorating into moderate to severe dementia, but they wouldn’t always recognise palliative signs, whereas the palliative nurses would have said we can recognise the palliative signs, but we wouldn’t know the dementia signs’ (Service commissioner 1)* Health care professionals attended either a Dementia Awareness training day or completed a Certificate Course in Holistic Dementia Care.
D11: Societal and Ethical Issues	Phase 3- Focus Group with Health care professionals Theme: Working in Partnership Phase 3- Interview with Health care professional Theme: Working in Partnership TIDieR (Supplemental Appendix 1) Description of the Hospice Enabled Dementia Partnership	**Equity of access to palliative care for people with dementia** There was a professional consensus on the importance of a partnership approach between mental health and palliative care services. Taking time to develop good relationships was pivotal to the success of the project: *‘The first year of the project, building those relationships was probably one of the most important things. . .there was a genuine willingness for each world of mental health and palliative care, wanting to get this right for this patient population. I think if it wasn’t for the project’s creation, we wouldn’t have the partnerships now that we have and we didn’t have at the beginning of the project’ (Health care professional 16)* **A reciprocal partnership relationship in cross working between mental health and palliative care services was generated and promoted by the project enabling equity of access to palliative care for this population:** *‘I think this project has been really invaluable in promoting the partnerships between ourselves and palliative care. People I have been worried about have been referred through and the project have been very, very quick to pick them up. Even just building personal relationships with people out of the hospice, it’s been very helpful and very positive. They know they can pick up the ‘phone and ring me if they want advice about something’ (Health care professional 31)* As described in the TIDieR the Dementia-Friendly Day Hospice with emphasis on building design, signage, lighting and colour contrast played a large part in equity of service access to people with dementia referred to the Hospice Enabled Dementia Partnership.

**Table 5.** Key Findings of the study.

Phase 1.
• 100 people with Dementia were referred to the project May 2016–December 2017 • 42 people (42%) died during the project (38 died at home and 4 in Hospital) • Family carers reported that preferred place of care and death was achieved for all 38 people (38%) who died at home • Advance care planning took place with 22 people (22%) with dementia and preference outcomes were recorded • Anticipatory prescribing for out of hours care took place with the majority of people referred
**Phase 2 and Phase 3 (4 Core Themes)**
Impact Of Dementia • Undiagnosed Pain • Aggression, agitation, depression and restlessness • Family carer burden and experience of loss (All of the above are relevant to services offered by Hospice Enabled Dementia Partnership)
**Value of the Service** • Dual holistic support and therapies to the person and family carers • Peer support for family carers at Day Hospice • Emotional support from staff • Benefits of therapies for patient observed by staff and family carers • Benefits of therapies for family carers • Person centred care • Patient isolation addressed • Symptom and behavioural assessment and management
**Information and Learning Needs** • All participants understood palliative care • Project highlighted applicability of palliative care to non-malignant disease • Family carers perceived staff to be competent and skilled • Staff identified other professional training needs • Some family carers had training on dementia care • Family carer learning needs were identified • Collaborative working generated shared learning and expertise between services • A Public Health Approach was promoted to raise awareness of palliative care and dementia • Modes of education were identified to disseminate knowledge from the Project **Working in Partnership** • Family carers reported that staff included them in decision making and care planning • Shared decision making and care planning enabled family carers to be partners in care with prior knowledge of the person with dementia • A reciprocal, partnership relationship in cross working between mental health and palliative care services was generated and promoted by the project

## Discussion

This study sought to evaluate a model of Hospice Enabled Dementia Partnership, mapped to international domains of best practice.^11^ A novel framework is presented, integrating the care of people with dementia and family carers within a hospice environment showing positive outcomes and impact, learning and development and broadening of networks for staff.

### Impact of dementia

Those referred had a range of dementia diagnoses and for some a co-morbidity. The range of symptoms, and impact of dementia on the person and family resonates with studies internationally^14,44–46^ and shows the relevance of the Hospice Enabled Dementia Partnership to this population and family carers. The impact and carer burden, reported by professionals, was similar to that documented across the world.^47–49^ Family caregivers were dealing with anticipatory grief due to the loss of the person as they knew him or her.^50^ They recognised a need to develop inner strength and sought to become resilient in their role.^51^

### Value of the service

All participants recognised the value of this service model. Family caregivers perceived a uniqueness about the Dementia-Friendly Day Hospice due to the dual holistic assessment, support and therapies for them and their family member. This weekly dual support was lacking within other Day Care facilities. Family caregivers accessed peer support and witnessed their loved ones responding positively to therapies. A sense of belonging and acceptance was perceived to help address isolation^52^ as the person participated in therapies or were assisted by staff. The benefits of this service were also apparent at home for those too unwell to attend Day Hospice.

Professionals showed enthusiasm and commitment to this new service. It enhanced job satisfaction by empowering them with skills and knowledge to assess and provide end-of-life care as services worked together in a joined-up care pathway. In addition to the dual benefits of therapies the project achieved a number of positive recorded outcomes such as achievement of preferred place of care for all 38 people who died during the project and anticipatory prescribing for out of hours care with the majority of people. The Clinical Lead of the service facilitated advance care planning with almost one quarter of people referred. Outcomes included power of attorney, right to refuse treatment and preferred place of care and death. The remainder of those referred to the project lacked capacity to take part in advance care planning and if required, best interest discussions took place. Earlier introduction of advance planning conversations in primary care, or with dementia services following diagnosis could have promoted more advance care planning taking place.^53^

### Information and learning needs

Both family carers and professionals reported unmet learning needs, most of which were identified and addressed through carer and professional education. Family carer unmet learning needs were similar to those within international literature.^27–30^ It is recognised that family carer’ information and education are an important supportive intervention.^54^ Family carers also believed they were learning from their practical experience of caring for someone with dementia and had a desire to develop their preparedness in this role. They recognised learning needs in advance care planning resonating with a randomised controlled trial, which found that advance care planning can reduce uncertainty in decision making for informal carers of people with dementia.^55^ All family carers perceived staff to have a good level of preparedness in knowledge and skills, whilst staff perceived they lacked competencies and identified areas for training. Key to professional education was thought to be the fusion of knowledge and skills from collaboration between palliative and mental health services. A wider Public Health Approach was proposed by participants to promote the concept of palliative care for people with dementia.^56^

#### Working in partnership

Collaborative practice across services has had a global emphasis,^57^involving ‘sharing’, ‘working together’ and ‘role awareness’.^58^ Although mental health and palliative care services lack a history of working collaboratively, partnership working, and relationship building was generated. Time invested in early discussions and joint service planning were key to this result. This centrality of partnership working between services was a thread throughout the data and enabled integrative coordination of care. Core to person centredness service users were at the heart of this partnership.^59,60^ Shared decision-making and care planning enabled family carers to be partners in care with prior knowledge of the person with dementia.^60,61^ Identified enablers and challenges to collaborative working resonate with a best practice model for partnership practice between palliative care and intellectual disability services,^60^ in relation to fusion of knowledge and skills, robust assessment, care planning and maintenance of place of care.^60^

### Strengths and weaknesses of the study

This study’s strength is the integration of views from the interdisciplinary care team, and family carers, enabling a broad spectrum of insights. Limitations are possible female gender bias and, lack of information on hospital admissions out of hours. The study involved healthcare systems in one region of the United Kingdom which may vary internationally.

#### What this study adds

This study provides novel insights into the delivery of a dementia- friendly partnership across hospice and community settings, the practicalities, and outcomes of delivering the service and the untapped potential such services have in ensuring that the person with dementia and carers are placed at the centre. Whilst the evaluation of this model provides initial evidence, more longitudinal studies on effectiveness and implementation are required.

## Conclusion

This study sought to evaluate a model of partnership, based on international domains of best practice, between a large specialist palliative care hospice provider, a dementia charity and a Health Care Trust. Positive outcomes and impact resulted, such as achievement of preferred place of care and death at home, dual benefits of therapies for patients and families and partnership in cross working and learning between services. This model should be replicated given the international relevance and incidence of dementia globally.

## Supplemental Material

sj-pdf-1-pmj-10.1177_02692163221116763 – Supplemental material for Evaluating a partnership model of hospice enabled dementia care: A three-phased monitoring, focus group and interview study Click here for additional data file. Supplemental material, sj-pdf-1-pmj-10.1177_02692163221116763 for Evaluating a partnership model of hospice enabled dementia care: A three-phased monitoring, focus group and interview study by Dorry McLaughlin, Felicity Hasson, Joanne Reid, Kevin Brazil, Lesley Rutherford, Carol Stone, Jenny T van der Steen and Joanne Ballentine in Palliative Medicine

sj-pdf-2-pmj-10.1177_02692163221116763 – Supplemental material for Evaluating a partnership model of hospice enabled dementia care: A three-phased monitoring, focus group and interview study Click here for additional data file. Supplemental material, sj-pdf-2-pmj-10.1177_02692163221116763 for Evaluating a partnership model of hospice enabled dementia care: A three-phased monitoring, focus group and interview study by Dorry McLaughlin, Felicity Hasson, Joanne Reid, Kevin Brazil, Lesley Rutherford, Carol Stone, Jenny T van der Steen and Joanne Ballentine in Palliative Medicine
