# Supplementary material for: Evaluating a partnership model of hospice enabled dementia care: A three-phased monitoring, focus group and interview study
Source: Palliat Med. 2022 Sep 5;36(9):1351–63. doi: 10.1177/02692163221116763 (PMC9606481; doi:10.1177/02692163221116763)
Supplement: sj-pdf-1-pmj-10.1177_02692163221116763 – Supplemental material for Evaluating a partnership model of hospice enabled dementia care: A three-phased monitoring, focus group and interview study [file sj-pdf-1-pmj-10.1177_02692163221116763.pdf]

APPENDIX 1

The TIDieR (Template for Intervention Description and Replication) Checklist (Hoffmann et al, 2014)

**ITEM NUMBER**	**ITEM**
1.Brief Name of Intervention	Hospice Enabled Dementia Partnership Project
2.Rationale/Theory Essential to Intervention	The Intervention (Hospice Enabled Dementia Partnership) was informed and underpinned by EAPC Domains of Optimal Palliative Care for People with Dementia (van der Seen et al, 2014). The care-related domains included: Applicability of palliative care, person centred care, communication and shared decision making, setting care goals and advance care planning, continuity of care, prognostication and timely recognition of dying, avoiding aggressive or futile treatment, optimal symptom management and providing comfort, psychosocial and spiritual support, family care and involvement, education of the healthcare team and societal and ethical issues.
3.What Materials Were Used?	The intervention providers developed enhanced knowledge and skills in palliative dementia care through education, shadowing colleagues from other disciplines and through partnership working between specialist palliative care and mental health services enabling reciprocal learning. Educational programmes, which the interventional providers attended, ranged from individual study days on Dementia Awareness to the European Certificate in Holistic Dementia Care- a 10 week, multi-disciplinary, distance learning course developed in collaboration with palliative care and mental health services. A number of different assessment tools were used such as: Distress and Discomfort Assessment Tool (DisDAT) and ABC Behaviour Chart. Also prognostication tools were used such as: the Supportive and Palliative Care Indicator Tool SPICT-2-sided.pdf (oxfordshireccg.nhs.uk). Family carers involved with the Hospice Enabled Dementia Partnership were able to attend the Dementia Charity Training for informal caregivers, received support from the intervention providers in relation to their role as Carers and financial planning/ legal support from the Dementia Charity advocate
4.What Procedures/Activities Were Used ?	Holistic assessment of the person with dementia, and family carer, with consideration to dementia concerns; care planning based on initial assessment; dual support to the person with dementia , and family carers in a Dementia-Friendly Day Hospice with emphasis on building design, signage, lighting and colour contrast; A bespoke creative therapy programme (involving music therapy, art therapy, reminiscence therapy and movement and music workshops); A bespoke complementary therapy programme(offered to people with dementia and their family carers in their own home or in the Dementia-Friendly Day Hospice); end of life and support at home for people with dementia (involving

	pain and symptom assessment and management and assessment of behaviours and of the environment in managing behaviours).
5.Who Provided the Intervention?	Intervention providers were:<br>1)<br>A Specialist Palliative Care Dementia Team (Project Lead and Dementia Day Hospice Team) who liaise with the Dementia-Friendly In-Patient Unit and Hospice Community Specialist Palliative Care Team as required.<br>2)<br>Partnership working with a Dementia Charity offering an advice and support service, Mental Health Services, Specialist Community Oncology and Palliative Care Team and Primary Care Services aligned with the local Health and Social Care Trust<br><br>The Hospice Enabled Dementia Partnership was guided by a Project Delivery Team, and a Project Steering Group, the latter comprising of a range of Health Care Professionals (with an interest in palliative and dementia care) and a Carer. The Project Steering Committee met every 3 months during the Project.
6.How Was the Intervention Provided?	The intervention was provided face to face individually (complementary therapy, emotional support/ financial advice to family carers, holistic individual assessment and care planning, which included advance care planning)<br><br>Other aspects of the intervention were provided in groups such as creative therapy/ music therapy and movement and complementary therapy, peer support to family carers in the Dementia-Friendly Day Hospice<br><br>Key to the delivery of these activities was partnership working across services involved with delivering the project and outlined in Item 5 above
7.Where did the Intervention Occur?	Dementia Day Hospice, Home Setting of Person with Dementia and Family Carer, Dementia-Friendly In-Patient Unit. This was a community-based project with only one person with dementia requiring In-Patient admission for symptom management associated with a co-morbidity
8.When and How Much was the Intervention Delivered?	One hundred people were referred to the Hospice Enabled Dementia Partnership project during the period May 2016-December 2017.

	The Referral Criteria for the Hospice Enabled Dementia Partnership for people living with a dementia were as follows:<br>• Unresolved, complex physical, emotional, social or spiritual symptoms and/or<br>• A need for specialist end-of-life support (for person with dementia and family caregiver) including care in the last days of life and bereavement services and/or<br>• A need for specialist palliative rehabilitation with clear functional goals and/or<br>• A need for a proposed period of respite provided by specialist palliative care in the case of emergency/crisis situations.
9.Tailoring: Was the Intervention Personalised, Titrated or Adapted- (What? Why, /When or How?)	The intervention was tailored around the holistic needs of the person with dementia and their family carers. These needs were determined through a holistic assessment.<br>Each **referral** received was triaged at point of delivery by the Project Lead to determine priority for response. Initial contact made via telephone with carer or patient, appropriateness for which was dependant on information received in the referral. This telephone contact was to discuss initial, paramount concerns and plan if a visit to the Dementia-Friendly Day Hospice was appropriate or if the patient required a home visit by the Project Lead as Hospice Nurse Specialist (Dementia). A 1st assessment appointment was agreed at this point. Reasons for a home visit would be that the patient was too unwell to travel to the Dementia-Friendly Day Hospice for appointment.<br>On the **1st assessment**, whether in the Dementia-Friendly Day Hospice or at home, a detailed history was taken by the Project Lead, from both patient and carer to establish a specialist palliative care plan for intervention. These interventions could be for physical, social, emotional or spiritual symptom management and support.<br>**Inter-professional team working** was an integral part of any intervention, with patients discussed at the weekly team meeting and onward referral to other disciplines such as social work, complementary therapist, occupational therapist and creative therapist, who were all part of the Day Hospice Dementia Team.

	**Symptom assessment and management** were carried out by the Project Lead/Hospice Nurse Specialist Dementia in conjunction with patient's General Practitioner including areas such as pain management, breathlessness, mobility issues, eating and drinking, elimination or sleep disturbance. Areas such as mood, interaction and symptoms re exacerbation of dementia would also be considered.<br>**Advanced care planning** was also introduced, by the Project Clinical Lead of the Hospice Enabled Dementia Partnership, as were consideration of preferred place of care/death and ceilings of care. Who was important to the patient in terms of support was established? Discussions regarding enduring power of attorney (EPA) was also introduced if appropriate. If the patient lacked cognitive capacity for these discussions and if EPA had not been previously activated, best interest decision making process was discussed with carer/family.<br>**Carers** were offered attendance at carer support group for guidance advice as a carer of someone with dementia were offered one to one support if required. **Bereavement needs** were considered for both patient and carer.<br>**Patients and carers' needs** were reassessed weekly to determine effectiveness of interventions or if there was a need to titrate or adapt treatment plan or introduce further treatment plans for new symptoms.
10.Modifications: If the Interventions was Modified Describe the Changes (What, why, When and How?)	Modifications for interventions were often required due to changes in the patient' condition caused by deterioration, exacerbation of behavioural symptoms causing carer distress or the patient's condition deteriorating and he/she requiring end of life care support.
11.How Well Planned? Adherence or Fidelity?	For the first year of the Hospice Enabled Dementia Partnership time was allocated for Service Leads and Stakeholders to meet and build relationships and friendships across mental health and palliative care services. This appeared to generate a joint willingness and commitment for service collaboration across sectors and for the project to be successful. Meetings with service leads and stakeholders continued as a Project Steering Group and Project Delivery Team.
12.How Well Actual? Was Intervention Delivered as Planned?	The intervention was delivered as planned but there was limited participation in the project by Mental Health Services during the timeframe of the evaluation. However, partnership and good relationship building did occur with those from Mental Health Services who were participating.
