# Supplementary material for: Evaluating a partnership model of hospice enabled dementia care: A three-phased monitoring, focus group and interview study
Source: Palliat Med. 2022 Sep 5;36(9):1351–63. doi: 10.1177/02692163221116763 (PMC9606481; doi:10.1177/02692163221116763)
Supplement: sj-pdf-2-pmj-10.1177_02692163221116763 – Supplemental material for Evaluating a partnership model of hospice enabled dementia care: A three-phased monitoring, focus group and interview study [file sj-pdf-2-pmj-10.1177_02692163221116763.pdf]

APPENDIX 2

Interview Guide for Family Carers' Interviews

Interview Guide

Can you tell me about your family member who has a diagnosis of Dementia? How long ago was this diagnosed? How was it diagnosed? How was this diagnosis given to you/ and your family member?

Were you provided with information about Dementia and the care and support which your family member and you could receive? How did you get this information? Was there any other information which you would have liked?

What do you understand about palliative care? Do you feel that palliative care should be part of the care and support which people with Dementia and their family members should be offered?

In relation to the Hospice Enabled Dementia Care offered by the Hospice which services has your family member been referred to- Community Palliative Care Team, Day Hospice, In-Patient Unit? What are your views on the Hospice Enabled Dementia Care which your family member is receiving from these services (Community Palliative Care Team/ Day Hospice/ In-Patient Unit)? What works well? Is there anything which you feel could be changed or improved?

How has Dementia affected your family member's life? Can you describe some of the symptoms which your family member has experienced due to Dementia? How have these symptoms been managed-Has your family member received care and treatment of symptoms to provide comfort?

Do you feel that services providing care and support in the Hospice Enabled Dementia Care work in partnership with you and your family member, where possible? If yes-How did they do this? Can you give an example of this joint working? Are you involved in decision making in relation to the care which your family member received and in setting goals for care/care planning? Has your family member been able to be involved in any of this decision making?

How do you feel you have been supported by the Hospice Enabled Dementia Care as a family carer of someone with Dementia? What support have you received and do you feel that your support needs have been met? Have you receive respite care in any form? Have you received psychological care and support? Spiritual care and

support? Financial advice in relation to benefits which your family member and/or you could access?

What thoughts do you have on the education and training which are needed for professionals caring for people with Dementia at end of life? What should this education and training involve? Do you feel that professionals providing the Hospice Enabled Dementia Care are well prepared and trained to care for and support both your family member and you? If no-how could they become better prepared and trained for this role?

Are there any other comments that you would like to make about the Hospice Enabled Dementia Care led by the Hospice?

Thank you for your time and for contributing to this interview. It is much appreciated. (Information and Support Pack to be given to the participant)

APPENDIX 2 (Continued)

Topic Guide for Focus Groups and Interviews with Health and Social Care Professionals

Focus Group/ Interview Guide

Can we start by you each telling me about your role in relation to Dementia Care Services/ Palliative Care Services?

What are your views on the relevance of palliative care to people with Dementia? Do you feel that palliative care should be a recognised part of the care and support that people with Dementia and family carers are offered? If Yes or No- Can you tell me more about this?

Can you tell me your views on Hospice Enabled Dementia Care? What do you think has worked well? Is there anything which you feel could be changed or improved? What have been the challenges? How have or could these challenges be addressed? Was there anything which would have hindered professionals from referring patients with Dementia to the Hospice Enabled Dementia Partnerships Project? How do you feel the principles of palliative care, for people with Dementia, could be more widely transferred to generalist palliative care and mental health services?

Can you describe how you, and your team, worked in partnership with the person with Dementia, where possible, and/or family members? Did this partnership involve shared decision making? Can you tell me how you and your team provide information about Dementia and palliative care to people with Dementia, where possible, and their family carers?

How does your team assess the holistic palliative care needs of the person with dementia throughout the trajectory and their family members? Is this normally an assessment by a multi-disciplinary team? Can you describe assessment tools that you use with people with Dementia?

What are the common symptoms which people with Dementia that you care for experience? How do you and your team address these symptoms? Can you describe holistic approaches that you use to treat symptoms and promote comfort?

Can you talk about some of the challenges which you and your team found in initiating advance care planning discussions with people with Dementia? In relation to people with Dementia that you have cared for to date were advance care planning discussions early in the disease process or on the whole was it necessary to take 'best interest decisions'?

Where you, and your team, were caring for someone with Dementia at home or in a nursing home did you aim to prevent unscheduled hospital admissions through regularly assessing and anticipating the person needs (eg: out of hours care, anticipatory prescribing of as needed medications, ensuring equipment available)? How did this help?

Do you feel that services providing palliative care and support to people with Dementia and family members work in partnership with each other? Can you describe how this

happens in your service? What do you feel helps partnership working between services in relation to palliative and end of life care for people with Dementia? What hinders partnership working between services?

What education and training did you have in relation to caring for people with Dementia at end of life and their family carers? What thoughts do you have on the education and training which are needed for professionals caring for people with Dementia at end of life? What should this education and training involve? How well prepared and trained did you feel that you are in providing Hospice Enabled Dementia Care to care for and support both people with Dementia and their family carers. If not well prepared and trained-how could you become better prepared and trained for this role?

Are there any other comments that anyone would like to make about Hospice Enabled Dementia Care or palliative and end of life care for people with Dementia generally?

Thank you for your time and for contributing to this interview. It is much appreciated.
(Information and Support Pack to be given to each participant)

APPENDIX 2 (Continued)

Interview Guide for Telephone Interviews with Service Commissioners

Interview Guide

Can you start by telling me about your role in relation to Dementia Care Services/ Palliative Care Services?

What are your views on the relevance of palliative care to people with Dementia?

Have you had involvement with the Hospice Enabled Dementia Partnerships Project which the Hospice is leading? What are your thoughts and perceptions of this model of care?

What are your views on the need for enhanced palliative and end of life care for people with Dementia? How do you feel the principles of palliative care for people with Dementia could be more widely transferred to generalist palliative care and mental health services?

How does this model of Hospice Enabled Dementia Care fit in with current statutory service provision? What hinders partnership working between services in relation to palliative and end of life care for people with Dementia? What do you feel helps partnership working between services in relation to palliative and end of life care for people with Dementia?

What do you see as the benefits of this model of care?

What has worked well? What are the enablers to the delivery and success of this model of care?

What are the challenges to the delivery and success of this model of care?

How important is it that this model of care is sustained/further developed? What is the strategic direction for this model of care?

Would you see this Hospice Enabled Dementia Care model as being transferable to other parts of UK/ Europe?

Is there anything else you wish to add –are there any other comments that you want to make in relation to that which we haven't talked about?

Thank you for your time and for contributing to this telephone interview. It is much appreciated. (Information and Support Pack to be forwarded)
